# Changes in spike protein antibody titer over 90 days after the second dose of SARS-CoV-2 vaccine in Japanese dialysis patients

**DOI:** 10.1186/s12879-022-07809-1

**Published:** 2022-11-14

**Authors:** Haruki Wakai, Natsumi Abe, Touno Tokuda, Rika Yamanaka, Satoshi Ebihara, Kensuke Izumaru, Daisuke Ishii, Toru Hyodo, Kazunari Yoshida

**Affiliations:** 1 Reiseikai Medical Corporation Shinagawa Garden Clinic, Imasu Ohsaki Building 2F, 1-20-3 Ohsaki, Shinagawa, Tokyo 141-0032 Japan; 2 Department of Urology, Kitasato University School of Medicine, 1-15-1 Kitasato, Minami-Ku, Sagamihara, Kanagawa 252-0374 Japan; 3 Reiseikai Medical Corporation Gotanda Garden Clinic, Kanpai Building 7F, 5-22-27 Higashi-Gotanda, Shinagawa, Tokyo 141-0022 Japan; 4 Reiseikai Medical Corporation Ebisu Garden Clinic, VORT Ebisu Maxim 9F, 3-9-19 Higashi, Shibuya, Tokyo 150-0011 Japan

**Keywords:** SARS-CoV-2, COVID-19, Vaccine, Dialysis, Antibody titer

## Abstract

**Objectives:**

There is no report on antibody titers after vaccination against SARS-CoV-2 in Japanese dialysis patients. As dialysis is different between Japan and other countries, changes in antibody titers were examined.

**Methods:**

Baseline characteristics and anti-spike protein antibody titers (Roche) over 90 days after administration of the BNT162b2 messenger RNA vaccine were investigated in dialysis patients.

**Results:**

The maximum anti-spike protein antibody titer after the second dose was 738 (327 to 1143) U/mL and was reached at 19 (17 to 24) days after the second dose. Antibody titers decreased over time, with titers of 770 (316 to 1089) U/mL at 15 days, 385 (203 to 690) U/mL at 30 days, 254 (138 to 423) U/mL at 60 days, and 208 (107 to 375) U/mL at 90 days after the second dose. When an antibody titer of 137 U/mL was assumed to be a measure related to breakthrough infection, the proportion of subjects with antibody titers exceeding this level was 90.1% at 15 days, 85.3% at 30 days, 75.0% at 60 days, and 65.4% at 90 days after the second dose. When a decrease in antibody titers below the assumed breakthrough level was defined as an event, subjects with a pre-dialysis albumin ≥ 3.5 g/dL were significantly less likely to experience an event than subjects with a pre-dialysis albumin < 3.5 g/dL.

**Conclusions:**

The presence of anti-spike protein levels ≥ 313 U/mL at 30 days after the second vaccine dose might be a factor in maintaining enough antibody titers at 90 days after. Whether an additional vaccine dose is needed should be determined based on indicators serving as factors in maintaining antibody titers as well as the status of the spread of infection.

**Supplementary Information:**

The online version contains supplementary material available at 10.1186/s12879-022-07809-1.

## Introduction

Dialysis patients have a higher risk of infections than non-dialysis patients [1–4]. Also, in Japan, it was reported that the mortality rate of infections among dialysis patients was approximately eight times higher than that in the general population [5]. In addition, a survey on Japanese dialysis patients in 2019 showed that 21.5% of the documented causes of death were caused by infections, the second most common cause of death after heart failure (22.7%) [6]. These reports highlight that infectious disease control among dialysis patients is an issue.

The environment surrounding dialysis patients in Japan has different aspects from that in other countries. It was reported that the rate of internal shunt creation in Japan is 91%, which is higher than the rates of 69% in Europe and 68% in the U.S. [7]. In addition, in Japan, catheters or artificial blood vessels, which carry a high risk for infection, are less likely to be used than in other countries [8]. Also, dialyzers are not reused, and the usage of intravenous irons is also lower than in other countries [9]. Robinson et al. reports that the risk of death among dialysis patients in other countries compared with Japan is 2.4-fold and 2.8-fold, respectively, in terms of one-year survival and five-year survival rates [10].

Vaccines against SARS-CoV-2 have been developed by various pharmaceutical companies, gained widespread use. Antibody titers induced by these vaccines have been already well-documented in the general population. Although there are several published reports on changes in vaccine antibody titers among dialysis patients outside of Japan [11–14], changes in vaccine antibody titers among dialysis patients in Japan may be different from those in other countries. Thus, we considered it significant to examine changes in antibody titers among Japanese dialysis patients.

As of October 23, 2021, among approximately 344,000 dialysis patients in Japan [6], only 2644 patients, approximately 0.8%, were infected [15]. A total of 412 (16%) dialysis patients have died after infection and 1056 patients (39.9%) had an unknown outcome [15]. We might say that strict control by the government, public, and medical institutions has led to favorable outcomes in both the prevention of the spread of infection and treatment after infection. Therefore, we add to data on antibody titers of vaccine to contribute to infection prevention.

## Materials and methods

### Ethic approval and consent to participate

This study was approved by the ethics committee of Reiseikai Medical Corporation (July 2nd, 2021) and conducted in accordance with the Declaration of Helsinki and the ethical guidelines provided by the Ministry of Health, Labour and Welfare. As this study involved no invasion or intervention to patients and used only information such as medical information and residual samples, an opt-out was provided to disclose information, and an opportunity for refusal was assured. And, all participants gave consent to participate after having been informed about the nature and purpose of the study. This study is registered with the Clinical Trials Registry (UMIN000046281).

### Study design

This study was a collaborative observational study at two centers. Patients were included in this study when they met all of the following inclusion criteria and did not meet any of the following exclusion criteria (with the rationales presented in parentheses).

#### Inclusion criteria

1. Hemo-dialysis patients who regularly visited the study center (in order to track data).
2. Patients who received the BNT162b2 messenger RNA vaccine (Pfizer-BioNTech) (hereinafter referred to as “BNT162b2 vaccine”) dose in June 2021 (to examine antibody titers induced by vaccination).

#### Exclusion criteria

1. Patients who have been infected with SARS-CoV-2 (to understand the actual situation in uninfected individuals because many Japanese people have not been infected). We excluded patients those who had a positive result by PCR test before the start of the study and those who had a positive result by antigen test. No initial screening tests were performed just for this study.
2. Patients who received only one vaccine dose (because antibody titers do not increase without two vaccine doses).

### Method

Anti-spike protein (Anti-S) IgM antibody titers (assay reagent: SARS-CoV-2 IgM assay reagent (IB), assay system: “Lumipulse® L2400” and “Lumipulse PrestoII,” FUJIREBIO Inc., Tokyo, Japan), anti-S IgG antibody titers (assay reagent: SARS-CoV-2 S-IgG assay reagent (IB), assay system: “Lumipulse® L2400” and “Lumipulse PrestoII,” FUJIREBIO Inc., Tokyo, Japan), and anti-spike protein antibody titers (Elecsys Anti-SARS-CoV-2 S antibody qualitative test reagent, Roche Diagnostics K.K., Tokyo, Japan) to SARS-CoV-2 were measured approximately once every two weeks after the first dose of the BNT162b2 vaccine. Antibody levels were measured using residual samples of the blood collected for routine tests at dialysis sessions. Anti-S IgM antibody titers (Fujirebio) were measured from 1 to 65 days after the first dose, Anti-S IgG antibody titers (Fujirebio) were measured from 1 to 76 days after the first dose. Anti-spike protein antibody titers (Roche) were measured from 22 to 95 days after the second dose. Although Fujirebio’s reagents were originally used for examining changes in both anti-S IgM antibody titers and anti-S IgG antibody titers, they were then replaced with Roche’s assay of anti-spike protein antibody titers for the following two reasons. One reason is that, although some samples showed undetectable anti-S IgG antibody titers as determined by Fujirebio’s assay, such measured values were required to be evaluated as specific values that were not below the limit of detection. The other reason is that the use of measured values obtained by Roche’s assay, which is widely used worldwide, provided comparability with other study results. Major results obtained from this study were expressed as anti-spike protein antibody titers obtained using Roche’s reagent.

Data with only anti-S IgG antibody titers available due to failure to test anti-spike protein antibody titers were imputated with values calculated using the approximation formula “anti-spike protein antibody titer (U/mL) = 9.7246 × anti-S IgG antibody titer (AU/mL) – 19.614.” (Additional file 1) This approximation is based on the data accumulated in this study. (Measured anti-S IgG antibody titers < 2.02 were observed in three patients at 15 days and two patients at 30 days and were all < 1. For Endpoint 3, these measured values were handled as 0.) Values calculated using the approximation formula were only used at 15 days and 30 days (All the data at 15 days, 31 cases of 95 cases at 30 days).

### Survey items

Baseline subject demographics included age, sex, underlying disease(s), status of comorbid diabetes, height, body weight (post-dialysis), Body Mass Index (BMI), history of dialysis, creatinine index (Additional file 2), C-reactive protein assay, HbA1c (NGSP), PTH-intact, glycated albumin, ferritin, geriatric nutritional risk index, Kt/V (Shinzato), protein catabolism rate, Transferrin saturation, albumin (pre- and post-dialysis) and clear space ratio. (Unless otherwise specified, the last pre-dialysis values before the first vaccine dose are shown.)

Anti-S IgM antibody titers, anti-S IgG antibody titers, and anti-spike protein antibody titers after vaccination were tested approximately once every two weeks after the first vaccine dose, coinciding with blood collection for routine tests, and values up to approximately 90 days were evaluated.

As this study was designed to examine antibody titers over a long period of time, these endpoints concern only this report, and the study continues further.

### Endpoints

The endpoints for this report are shown below:

1. Antibody titers after the second dose
2. Factors affecting anti-spike protein antibody titers, and contributing to anti-spike protein antibody titers > 137 U/mL at 90 days after the second dose (Chloe’s report indicated that the protection was 89.3% with antibody titers > 141BAU and 12.4% with antibody titers of 13 to 141BAU [16]. This antibody titer of 141 BAU corresponding to 89.3% protection was converted to 141*0.972BAU≒137 U/mL using the conversion formula based on the report by Krzysztof et al. [17], which was assumed as a measure related to breakthrough infection. An analysis was performed based on whether this level was exceeded. An antibody titer of 137 U/mL is hereinafter referred to as the assumed breakthrough level.)
3. Predictive performance of anti-spike protein antibody titers at 30 days for anti-spike protein antibody titers at 90 days exceeding the assumed breakthrough level
4. Correlation between anti-S IgM antibody titers and anti-S IgG antibody titers
5. Maximum anti-S IgM antibody titers and the number of days until the day with the maximum antibody

### Statistical analyses

Data analysis was performed using R version 3.5.2 (R Foundation for Statistical Computing, Vienna, Austria [https://www.R-project.org/]) and for Excel version 3.21 (Social Survey Research Information Co., Ltd., Tokyo, Japan). In the analyses of factors affecting anti-spike protein antibody titers (maximum values and values at 90 days), correlations between anti-spike protein antibody titers and various measured values were tested using Spearman’s test, and differences in anti-spike protein antibody titers between two groups defined by nominal variables were tested using the Wilcoxon rank sum test. In addition, in the multiple regression analyses including anti-spike protein antibody titers as an object variable, the normality of residual errors in the multiple regression analyses was tested using the Shapiro–Wilk test to determine its appropriateness. In the analyses of factors contributing to anti-spike protein antibody titers > 137 U/mL at 90 days, the Student’s t-test and Chi-squared test were used for continuous variables and nominal variables, respectively, in univariate analysis, while the Wald Chi-squared test was used in multiple logistic regression analysis. Correlations between anti-S IgM antibody titers and anti-S IgG antibody titers and between anti-S IgG antibody titers and anti-spike protein antibody titers were tested using Spearman’s test. As the latter was also used for conversion using an approximation formula, Pearson’s test correlation coefficient was also verified. In preparing a Kaplan–Meier curve, comparisons were made using the log-rank test and generalized Wilcoxon test. A two-sided significance level of 5% was used, and the analytical results in text were expressed as mean ± standard deviation or median (1st quartile to 3rd quartile).

## Results

Baseline subject demographics are shown in Table 1. A total of 96 patients met the eligibility criteria. They had an age of 68.5 ± 15.2 years (70.5 [57 to 81.25] years), consisted of 72 men (75%) and 24 women (25%), and had a duration of dialysis of 5.4 ± 5.3 years. There were no patients who use of immunosuppression.

**Table 1:** Participant characteristics (n = 96)

Item	Value
Age (years)	68.5 ± 15.2
Male/female	72(75%)/24(25%)
Duration of dialysis (years)	5.4 ± 5.3
Number of days to the second dose (days)	21.3 ± 2.5
Underlying disease	
Diabetes	40 (41.7%)
Nephrosclerosis	25 (26%)
Chronic glomerulonephritis	16 (16.7%)
Other	15 (15.6%)
Height (cm)	163 ± 10.1
Body weight (kg)	62.1 ± 16.5
Body Mass Index (kg/m^2^)	23.3 ± 5.9
Creatinine Index	90.1 ± 26.3
C-reactive protein (mg/dL)	0.45 ± 1.21
HbA1c (NGSP) (%)	6.5 ± 1.5
PTH-intact (pg/mL)	210.5 ± 221.1
Glycated albumin (%)	21.1 ± 4.8
Ferritin (ng/mL)	181.3 ± 174.6
Geriatric Nutritional Risk Index	93.6 ± 7.2
Kt/V (Shinzato)	1.41 ± 0.28
Protein catabolism rate(g/kg/day)	0.83 ± 0.15
Transferrin saturation (%)	28.3 ± 15
Pre-dialysis albumin (g/dL)	3.7 ± 0.4
Post-dialysis albumin (g/dL)	4.3 ± 0.6
Clear space ratio (%)	65.47 ± 7.44

n = 44 only for HbA1c and glycated albumin

Data are mean ± sd or n (%)

Unless otherwise specified, the last pre-dialysis values before the first vaccine dose are shown

### Antibody titers after the second dose

**Fig. 1:**
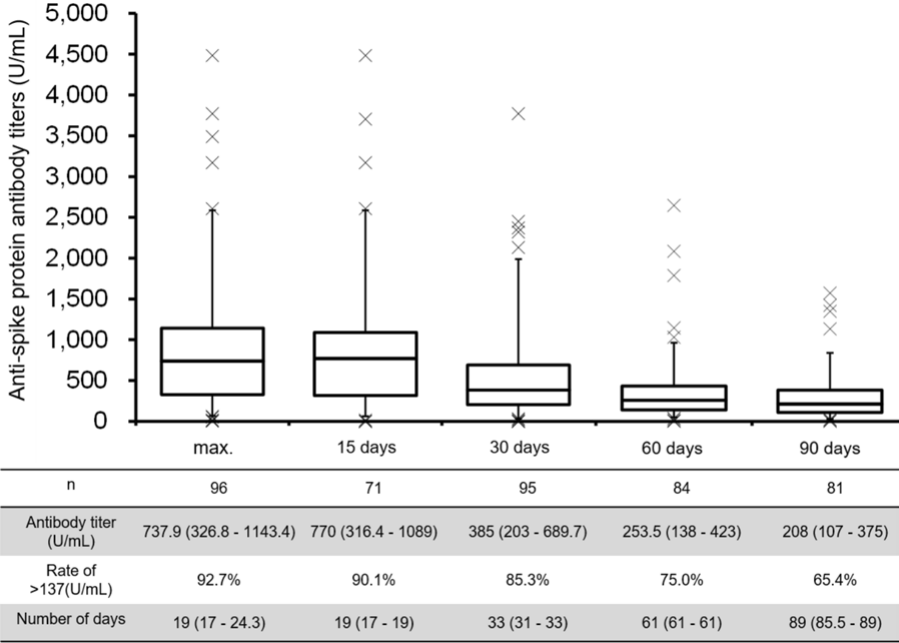
Changes in spike protein antibody titer from the day of the second dose (Roche). Data are presented by 5%-1st.Q-Median-3rd.Q-95%

Figure 1 shows the maximum anti-spike protein antibody titers after the second vaccine dose, and anti-spike protein antibody titers at 15 (± 4), 30 (± 10), 60 (± 10), and 90 (± 10) days after the second vaccine dose. The maximum anti-spike protein antibody titer after the second dose was 738 (327 to 1143) and was reached at 19 (17 to 24.3) days after the second dose, with two patients showing a persistent increase in antibody titers until the last day of measurement, including one patient who showed no significant increase in anti-spike protein antibody titers. Anti-spike protein antibody titers decreased over time, with titers of 770 (316 to 1089) at 15 days, 385 (203 to 690) at 30 days, 254 (138 to 423) at 60 days, and 208 (107 to 375) at 90 days after the second dose. The proportion of patients showing titers exceeding the assumed breakthrough level was 90.1% at 15 days, 85.3% at 30 days, 75.0% at 60 days, and 65.4% at 90 days after the second dose. Changes in anti-S IgG antibody titers are shown in Additional file 3a, b. In the majority of subjects, anti-S IgG antibody titers were undetectable after the first dose and before the second dose, generally peaked at three weeks after the second dose, and then declined. In addition, three patients still showed undetectable anti-S IgG antibody titers after the second dose, but had measurable, although very low, anti-spike protein antibody titers when tested using the same samples.

### Factors affecting anti-spike protein antibody titers

The results of the analyses of factors affecting maximum anti-spike protein antibody titers and antibody titers at 90 days after the second vaccine dose are shown in Additional files 4 and 5. A multiple regression analysis was performed, but with no normal distribution of residual errors obtained. Thus, this multiple regression analysis was not appropriate. The univariate analysis showed that high pre-vaccination creatinine index, high pre-vaccination pre-dialysis albumin, and an age younger than 70 years were the significant factors associated with high antibody titers (Additional file 4a, b).

**Fig. 2:**
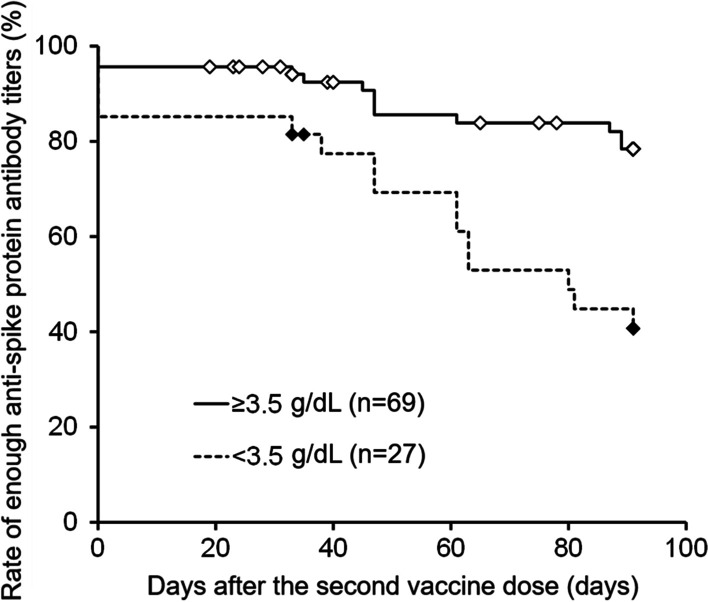
Kaplan–Meier curve for a spike protein antibody titer ≤ 137 as an event (comparison of albumin values ≥ 3.5 g/dL versus < 3.5 g/dL). An antibody titer of 137 U/mL was assumed to be enough. Censored cases were shown as diamond-shaped marks. p < 0.05 log-rank test, p < 0.05 for generalized Wilcoxon test

The univariate analysis of factors associated with anti-spike protein antibody titers at 90 days after the second vaccine dose exceeding the assumed breakthrough level showed that high pre-dialysis albumin and a young age were the significant factors (Table 2). The multiple logistic regression analysis with these two factors as well as sex, BMI, and history of dialysis as the explanatory variables showed that high pre-dialysis albumin and a young age were the significant factors, consistent with the univariate analysis (Table 3). In addition, in light of these results, a Kaplan–Meier curve using an anti-spike protein antibody titer below 137 U/mL as an event in the two groups defined by pre-dialysis albumins of ≥ 3.5/ < 3.5 was created and showed that the group with pre-dialysis albumins ≥ 3.5 was less likely to experience an event (Fig. 2).

**Table 2:** Factorial analysis of spike protein antibody titers > 137 at 90 days (univariate analysis)

Item	> 137 (n = 53)	≤ 137 (n = 28)	p
Height (cm)	163.2 ± 8.8	161.3 ± 12.6	0.411
Body weight (kg)	61.5 ± 16.4	61.1 ± 15.5	0.911
Body Mass Index (kg/m^2^)	23 ± 5.6	23.6 ± 6.5	0.666
History of dialysis (years)	5.4 ± 4.7	3.7 ± 2.8	0.085
Creatinine Index	93.6 ± 24.1	85.9 ± 25.8	0.185
C-reactive protein (mg/dL)	0.5 ± 1.5	0.3 ± 0.4	0.571
HbA1c (NGSP) (%) ^note^	6.5 ± 1.4	6.3 ± 1.6	0.615
PTH-intact (pg/mL)	194 ± 125.6	172.9 ± 101.8	0.447
Glycated albumin (%) ^note^	20.7 ± 3.9	21.9 ± 6.5	0.493
Ferritin (ng/mL)	184.3 ± 186.3	190 ± 167.7	0.893
Geriatric Nutritional Risk Index	94.1 ± 7.3	92.1 ± 7.8	0.248
Kt/V (shinzato)	1.4 ± 0.2	1.4 ± 0.3	0.230
Protein catabolism rate (g/kg/day)	0.9 ± 0.1	0.8 ± 0.2	0.340
Transferrin saturation (%)	29.3 ± 16.8	28.9 ± 13.5	0.896
Pre-dialysis albumin (g/dL)	3.7 ± 0.3	3.5 ± 0.4	0.013 *
Clear space ratio (%)	66.6 ± 6.4	64.4 ± 8.1	0.181
Post-dialysis albumin (g/dL)	4.4 ± 0.6	4.2 ± 0.6	0.131
Age (years)	65.7 ± 14.9	73.8 ± 14.1	0.020 *
Sex (Male/Female)	39 (73.6%)/14 (26.4%)	21 (75%)/7 (25%)	0.890
Diabetes	19 (35.8%)	13 (46.4%)	0.354
Nephrosclerosis	15 (28.3%)	9 (32.1%)	0.719
Chronic glomerulonephritis	10 (18.9%)	2 (7.1%)	0.158

Note: n = 22(> 137), n = 14(≤ 137)

^*^p < 0.05 Student’s t-test

(Unless otherwise specified, the last pre-dialysis values before the first vaccine dose are shown.)

**Table 3:** Factorial analysis of spike protein antibody titers > 137 at 90 days (multiple logistic regression analysis)

Variables	Odds ratio	95%CI	p
Sex (1: male, 0: female)	0.677	0.227–2.018	0.484
Body Mass Index (kg/m^2^)	0.961	0.886–1.042	0.337
Duration of dialysis (years)	1.087	0.939–1.258	0.266
Pre-dialysis albumin (g/dL)	2.855	1.243–6.558	0.013 *
Age (years)	0.967	0.941–0.995	0.019 *

Conditions causing titers > 137: High albumin values, younger age

Accuracy of the regression formula: Adjusted R2 = 0.170

Significance of the regression formula: p = 0.002

^*^p < 0.05 Wald chi-squared test

The predictive performance of anti-spike protein antibody titers at 30 days after the second vaccine dose for anti-spike protein antibody titers at 90 days after the second vaccine dose exceeding the assumed breakthrough level.

It was analyzed using a receiver operating characteristic (ROC) curve. Results showed that the cut-off value was 312.0 U/mL, and the area under the curve (AUC) was 0.921 (Additional file 6).

### The correlation between anti-S IgM antibody titers and anti-S IgG antibody titers

The correlation coefficient at each time point was ρ = 0674 (p < 0.001, Additional file 7a), and a significant positive correlation was observed with a correlation coefficient for the maximum value of ρ = 0.458 (p < 0.001, Additional file 7b) for each patient.

### The maximum anti-S IgM antibody titer

It was 1 (0.5 to 2.3) and was reached at 17 (7 to 19) days after the second vaccine dose.

## Discussion

### Comparison with reports on Japanese individuals without dialysis (30 and 90 days after the second vaccine dose)

This study showed that the anti-spike protein antibody titers among Japanese dialysis patients were 385 (203 to 690) U/mL at 30 days and 208 (107 to 375) U/mL at 90 days after the second vaccine dose, which were lower at both time points than the reports based on data from Japanese individuals without dialysis. A study of 2015 subjects previously reported that the median anti-spike protein antibody titer at 30 days was 2,060 U/mL [18], which represented an approximately threefold difference from the median maximum titer of 738 U/mL observed in individual subjects up to 30 days after the second vaccine dose in this study. In addition, the antibody titers at 90 days after the second vaccine dose have been reported to be 764 (423 to 1140) U/mL in a study of 378 subjects [19], which also represented an approximately threefold difference from the result from this study. In above two studies, they used the same assay reagents (Roche) as used in our study. This study suggested that anti-spike protein antibody titers among dialysis patients were lower than those among individuals without dialysis both at 30 days after the second vaccine dose and during the subsequent phase of decline. Hemodialysis patients were previously reported to have a reduced immune response [20], supported by our results. It is also reported that Japanese individuals without dialysis show a decrease in the median antibody titer to 28.6% between two weeks and three months after the second dose when using FUJIFILM’s test reagent [21]. Although the schedule was not strictly managed due to the observational nature of this study, a comparison with the value at 15 ± 4 days shows a decrease to 26.9%, suggesting that a percent decrease is almost the same with and without dialysis.

### Comparison with overseas reports on dialysis patients (around 30 days after the second dose)

A Germany study report using the same anti-spike protein antibody titer assay reagents as used in this study showed that anti-spike protein antibody titers were significantly lower in subjects with dialysis (253.5 U/mL) than in subjects without dialysis (1756 U/mL) [11]. These values in the Germany report are slightly lower than the antibody titers in this study. This is likely attributed to older age in the Germany study [75 (64 to 82) years] compared to this study [70.5 (57 to 81.25) years].

A French study of 69 subjects using the same reagent showed that the anti-spike protein antibody titers after the second dose were 284 (83 to 1190) U/mL [12]. Although these values are slightly lower than those in this study, no specific subject demographic variable explaining this difference was identified. A difference in the environment surrounding dialysis could contribute to this difference.

A study using Abbott’s reagent [13] reported the antibody titer of 276 AU/mL (IQR: 83.4 to 526.6) in dialysis patients compared with the antibody titer of 1082 AU/mL (735.0 to 1662.0) in individuals without dialysis, representing an approximately threefold difference, as observed in our study. Since the result of this study is consistent with our data, it may support our conclusion.

A Germany study comparing dialysis patients and individuals without dialysis using DiaSorin's reagent (for anti-S IgG antibody titers) reported that individuals younger than 60 years old, even with dialysis, showed the same level of antibody titers as that in individuals without dialysis [14]. In their report, differences are also likely to have arisen due to the upper limit of detection of the test reagent. It is likely that different reagents result in slightly different analytical results.

### Factors predisposed to an increase in antibody titers

Previous studies on dialysis patients have also previously reported that high albumin is a factor associated with high antibody titers [12, 13, 22], which was also confirmed in Japanese dialysis patients by our study.

Younger age has also been previously reported to be associated with higher antibody titers [12, 13, 23]. This study analyzed the subgroups defined by age (≥ 60/ < 60 years, ≥ 65/ < 65 years, ≥ 70/ < 70 years, and ≥ 75/ < 75 years) and found a significant difference in the comparison of ages ≥ 70 years and < 70 years, but the threshold in determining whether there is a significant difference may affect the population. However, as tests of correlation also showed a tendency toward negative correlations, it is considered sure that antibody titers are less likely to increase among older individuals.

There is no previous report on creatinine index and anti-spike protein antibody titers. Creatinine index reflects muscle mass. As muscle mass decreases, the body fluid compartment decreases, resulting in violent fluctuations in circulating blood volume associated with changes in total body water. In recent years, a modified creatinine index [24], a simplified version of the creatinine index, which is calculated by only age, sex, pre-dialysis serum creatinine level, and Kt/V for urea, has been proposed, and Arase et al. reported that a low modified creatinine index was associated with increased long-term risk of infection-related mortality in dialysis patients [25]. A larger skeletal muscle mass is associated with a higher creatinine index, and skeletal muscles have multilateral functions on immunity. Skeletal muscles are the most important organ that induces heat production [26] and play a key role in energy storage [27]. When acquiring an infection, individuals experience frequent muscle contractions, mainly during tremors, to increase body temperature for enhancing immunity [28]. Thermotherapy reduces viral and bacterial growth while promoting the migration and function of leukocytes and macrophages. A decrease in skeletal muscle brings about inadequate heat production and energy consumption, potentially causing an increase in the severity of infections.

Other reported factors leading to a predisposition to an increase in antibody titers among dialysis patients include previous infection [11, 23], duration of dialysis of < 5 years [22], high Kt/V [13], absence of the use of immunosuppressants, high white blood cell counts, and high gamma globulin levels [12]. Although these factors did not affect maximum anti-spike protein antibody titers in this study, different results may be obtained depending on the characteristics of the population.

Reported factors associated with high antibody titers among individuals undergoing dialysis include a young age [18, 21, 29], female sex [18, 21], previous infection with SARS-CoV-2 [18, 30], absence of comorbid diabetes [31], intervals of 18 to 25 days between the first and second doses, current intake of anti-allergic agents, absence of the intake of immunosuppressants/steroids, and absence of a drinking habit [18]. Regarding gender, given that males accounted for 75% of the subject population in this study, it is difficult to make an accurate comparison unless the ratio of females is increased. Other factors will be reviewed in further reports published in various countries or regions.

### Factors in maintaining antibody titers that are not expected to cause breakthrough infection

Reports on individuals other than dialysis patients describe factors that maintain high antibody titers that include a young age [19, 32], the absence of smoking [19, 32], female sex [32], and previous infection [33]. This study failed to identify significant factors associated with high anti-spike protein antibody titers at 90 days. Due to large variability in antibody titers, it is considered difficult to make evaluations using absolute values. In contrast, higher antibody titers than necessary are not required if antibody titers associated with breakthrough infection are maintained. Thus, the value corresponding to 89.3% protection as reported by Chloe [16] was converted to the antibody titer in this study [17], and an antibody titer of 137U/mL (assumed breakthrough level) was assumed as a measure related to breakthrough infection. Factors associated with antibody titers exceeding this level at 90 days were analyzed. Both univariate and multivariate analyses showed that high albumin and a young age were identified as the factors associated with anti-spike protein antibody titers exceeding the assumed breakthrough level. Although the mechanism underlying the effects of these factors is the same as discussed for the factors associated with increased maximum antibody titers, the Kaplan–Meier curve prepared as an explanatory analysis comparing albumin levels ≥ and < 3.5 suggested that the presence of albumin levels ≥ 3.5 might be an indicator of antibody titers kept above the assumed breakthrough level.

For the BNT162b2 vaccine, a study evaluating anti-SAb IgG antibody titers using Siemens’ reagent in dialysis patients reported that 8.1% (19/235) of subjects with an antibody titer ≥ 20 U/L (the upper limit of assay of Siemens’ reagent) at two months after vaccination had undetectable antibody titers by 180 days, whereas 61.1% (80/131) of subjects with the first measured antibody titer < 20 U/L had undetectable antibody titers by 180 days [34], suggesting that anti-spike protein antibody titers at 90 days may exceed the assumed breakthrough level when anti-spike protein antibody titers at 30 days were not less than 313 U/mL. Although repeated measurements of vaccine antibody titers cause the burdens of expense and blood collection, such burdens will be reduced if a measurement at 30 days estimates subsequent maintenance.

Although this study used Chloe’s report, there is no definitive report on antibody titers to prevent breakthrough infection. There are reports indicating that breakthrough infection occurred despite high neutralizing titers being maintained [35, 36]. The use of different test reagents between the studies makes it difficult to make comparisons, and potential mutations of the virus make it impossible to determine what level of antibody titers is adequate. However, currently available data may guide decision making.

In a six-month study in 1567 dialysis patients using Siemens’ reagent, anti-S IgG antibodies to the Ad26.COV2. S vaccine (Janssen Pharmaceutical K.K.) remained low from the beginning, median antibody titers to the BNT162b2 vaccine were decreased to approximately 1/6 at 6 months, and antibody titers to mRNA-1273 (Moderna Inc.) remained higher [34]. As our study included only individuals who received the BNT162b2 vaccine, caution should be exercised because the persistence of antibody titers varies with the type of vaccine. As our study also has only the ability to predict antibody titers up to 90 days after the second vaccine dose, further study is warranted to examine more long-term maintenance of antibody titers.

In addition, all is not lost in the event of a breakthrough infection. There is a previous report that patients with breakthrough infections all had mild cases of the disease [35]. Although, as a matter of course, some reports indicated that cases other than mild ones were observed [36], memory T-cells that persist in the body may enhance immune response associated with infection. Although this is only hypothetical because of the lack of currently available methods of measurement of memory T-cells, everything is not determined exclusively by vaccine antibody titers.

### About the new Omicron variant

Omicron escapes neutralization from antisera of vaccinated or convalescent individuals by ∼15-fold [37]. Some reports indicate that high titers of NAbs elicited by currently available mRNA vaccines do not protect against infection with the Omicron variant [38, 39]. However, boosting increased neutralization titers against Omicron and restored neutralization in all subjects compared to before boosting [37]. Some studies have shown that a third-dose of existing mRNA vaccines is still effective against infection with Delta and Omicron variants and hospitalization [40, 41]. And the third vaccine dose was effective in decreasing the risk of SARS CoV-2 infection during the Omicron wave compared with the second dose [42]. It's just not as impactful as it used to be, but it's still effective enough. Due to virus mutation, the meaning of antibody titers changes. Our conclusions will not apply forever. However, epidemiological data about changes in mRNA vaccine antibody titers in dialysis patients in Japan will serve as a reference for future vaccines against infectious diseases.

### Anti-S IgM antibody titers and anti-S IgG antibody titers

Anti-S IgM antibody titers reached a peak slightly earlier than anti-S IgG antibody titers. In addition, there was a significant positive correlation between both types of antibody titers at each time point. Moreover, tests of correlations were performed using maximum values from individual subjects in consideration of a gap in timing with maximum values, and again demonstrated a significant positive correlation. In contrast, three subjects with undetectable anti-S IgG antibody titers also showed detectable anti-S IgM antibody levels (ranging from 0.1 to 0.2). Although there is a correlation between anti-S IgM antibody titers and anti-S IgG antibody titers, this study suggested that some subjects showed no IgG response.

### Subjects with no increase in IgG antibody titers

As described above, three subjects had no increase in anti-S IgG antibody titers. A French study on dialysis patients previously reported that three of 69 patients showed undetectable anti-spike protein antibody titers after the second vaccine dose [12]. However, their report indicated that such undetectable antibody titers were attributable to previous immunological diseases. In addition, one of the three patients had an increase in antibody titers with the third vaccine dose. There are other reports that also indicated that patients who received a transplant using immunosuppressants did not develop antibodies [43]. In contrast, there is also a report that some patients showed no increase in antibody titers even in the absence of immunological diseases or use of immunosuppressants [44]. In our study, the T-cell response test (“T-SPOT® Discovery SARS-CoV-2”, RIKEN GENESIS CO., LTD., Tokyo, Japan) was performed on three subjects who never had an increase in anti-S IgG antibody titers, but two of the three subjects had no response and the remaining subject had almost no response. It should be noted that some subjects were unresponsive and inactivated. In addition, a comparison with the other subjects identified no specific laboratory parameters or patient demographics. These three patients will be followed up for a longer period of time and closely monitored for responses if they receive the third vaccine dose.

### Limitation

This study has several limitations.

One of them is that N antibodies were measured in not all samples before vaccination. Thus, in consideration of potential subclinical infection, nine subjects with anti-S IgG antibody titers not below the limit of detection before the second dose were tested for N antibodies approximately four months after the first vaccine dose, and all nine were found to be negative. However, the possibility cannot be ruled out that N antibodies became undetectable after vaccination. Thus, an additional analysis excluding these nine subjects was performed, but the results were not significantly different. Although the Roche’s test kit was not used in the initial stage of this study, the presence of converted values is another limitation. In this respect, both analyses including and not including converted values were performed for examination and demonstrated similar results (data not shown). In addition, as this study was an observational study using residual samples, tests were not strictly scheduled. Thus, for analysis, an appropriate period was set, and data closest to the reference date in the period were included, but data from some patients were outside the appropriate period, resulting in missing data. In addition, this study used the value corresponding to 89.3% protection based on Chloe’s report as the assumed breakthrough level, but this assumed breakthrough level is not an absolute value but just a reference value. It should be noted that this level is positioned as only a current guide of SARS-CoV-2, which will undergo further mutation. As this study was performed at two centers in Tokyo, Japan, our results cannot be uniformly applied to different environments, such as regions and equipment used at centers.

## Conclusions

The maximum antibody titers and the antibody titers at 90 days after the second vaccine dose in Japanese dialysis patients were 738 (327 to 1143) U/mL, and 208 (107 to 375) U/mL at 90 days after the second dose. The maximum antibody titers were approximately 30% of those in non-dialysis individuals reported elsewhere, and was similar to those in dialysis patients in other countries. The antibody titers at 30 days after the second vaccine dose might be a factor in maintaining enough antibody titers at 90 days after. As dialysis patients have lower antibody titers, it may be necessary for them to receive the third vaccine dose early. Whether an additional vaccine dose is needed should be determined based on antibody titers at 30 days, factors that maintain antibody titers, as well as indicators such as albumin and age, and the status of infection spread/reduction.

## Supplementary Information

**Additional file 1. **Correlation between ant-S IgG antibody titers (Fujirebio) and Anti-spike protein antibody titers (Roche).

**Additional file 2. **Calculation Formula for Creatinine Index.

**Additional file 3. **a. Changes in anti-S IgG antibody titers from the day of the first dose. b. Changes in anti-S IgG antibody titers from the day of the second dose.

**Additional file 4. **a Correlation with maximum spike protein antibody titers. b. Maximum spike protein antibody titers by baseline patient characteristics. b. Maximum spike protein antibody titers by baseline patient characteristics. c. Factorial analysis of maximum spike protein antibody titers. d. Factorial analysis of log-maximum spike protein antibody titers.

**Additional file 5. **a. Correlation with spike protein antibody titers at 90 days. b. Spike protein antibody titers at 90 days by baseline patient characteristics. c. Factorial analysis of spike protein antibody titers at 90 days.

**Additional file 6. **Predictive performance of spike protein antibody titers at 30 days for spike protein antibody titers >137 at 90 days.

**Additional file 7. **a. Correlation between anti-S IgM antibody titers and anti-S IgG antibody titers. b. Correlation between anti-S IgM antibody titers and anti-S IgG antibody titers (correlation between the maximums of both titers).

## Data Availability

The biochemical data used to support the findings of this study are based belong to the study team. For confidentiality reasons, the datasets are not publicly available. However, the data sets can be availed upon reasonable request from the corresponding author and with permission from the study team.

## Abbreviations

COVID-19: Coronavirus disease 2019
SARS-CoV-2: Severe acute respiratory syndrome coronavirus 2
Anti-S: Anti-spike protein
BMI: Body Mass Index
ROC: Receiver operating characteristic
AUC: Area under the curve
