# Supplementary material for: Changes in spike protein antibody titer over 90 days after the second dose of SARS-CoV-2 vaccine in Japanese dialysis patients
Source: BMC Infect Dis. 2022 Nov 14;22:852. doi: 10.1186/s12879-022-07809-1 (PMC9661455; doi:10.1186/s12879-022-07809-1)
Supplement: Supplementary file 1 — Additional file 1. Correlation between ant-S IgG antibody titers (Fujirebio) and Anti-spike protein antibody titers (Roche). [file 12879_2022_7809_MOESM1_ESM.docx]

Additional file 1. Correlation between ant-S IgG antibody titers (Fujirebio) and Anti-spike protein antibody titers (Roche)


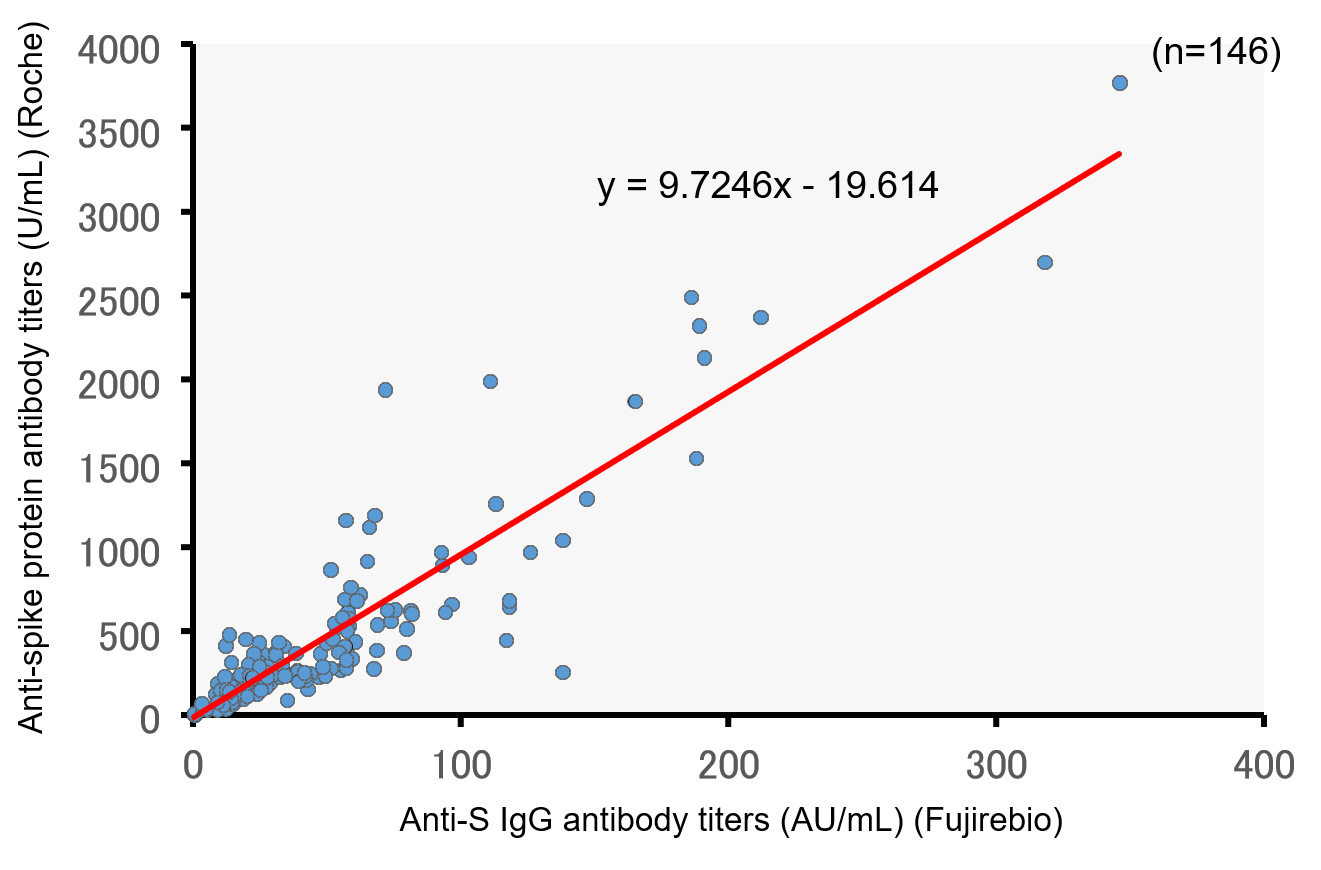


ρ=0.864, p<0.001 Spearman’s test.

ρ=0.900, p<0.001 Pearson’s test.

The results from the parametric test are also indicated due to conversion based on the derived linear regression formula.
