# Supplementary material for: Changes in spike protein antibody titer over 90 days after the second dose of SARS-CoV-2 vaccine in Japanese dialysis patients
Source: BMC Infect Dis. 2022 Nov 14;22:852. doi: 10.1186/s12879-022-07809-1 (PMC9661455; doi:10.1186/s12879-022-07809-1)
Supplement: Supplementary file 2 — Additional file 2. Calculation Formula for Creatinine Index. [file 12879_2022_7809_MOESM2_ESM.docx]

Additional file 2. Calculation Formula for Creatinine Index

Creatinine Index =Int (CRIND*10+0.5)/10

CRIND=GG/SG*100

SG=23.5-0.15*Age (male)

SG=19.6-0.12*Age (Female)

GG=GC*60*24 -(3.49*II-0.32)

GC=GCC*RVC

RVC=0.49

GCC=(G+(BW1-BW2)/VC*CRA/TIE) *1000

BW1=Pre BW *1000

BW2：Post BW *1000

Pre BW: Pre-Dialysis Body-Weight

Post BW: Post-Dialysis Body-Weight

VC=BW2×RVC

CRA=Pre CRE/100

Pre CRE: Pre-Dialysis Creatinine

TIE：72*60-TD

TD: Dialysis Time

II=0.96*(9.35*RVU*GU+0.29*RVU) +0.07

RVU=0.5538

GU=(G+(BW1-BW2)/VU*BUNA/TIE) *1000

VU：BW2*RVU

G=(BUNB-BUNA*Exp(-KTV)) *KTV/TD/(1-Exp(-KTV))

BUNA=Pre BUN/100

BUNB=Post BUN/100

BUN: Blood Urea Nitrogen

KTV: single-pool Kt/V (Shinzato)
