# Supplementary material for: Changes in spike protein antibody titer over 90 days after the second dose of SARS-CoV-2 vaccine in Japanese dialysis patients
Source: BMC Infect Dis. 2022 Nov 14;22:852. doi: 10.1186/s12879-022-07809-1 (PMC9661455; doi:10.1186/s12879-022-07809-1)

Additional file 3-a. Changes in anti-S IgG antibody titers from the day of the first dose


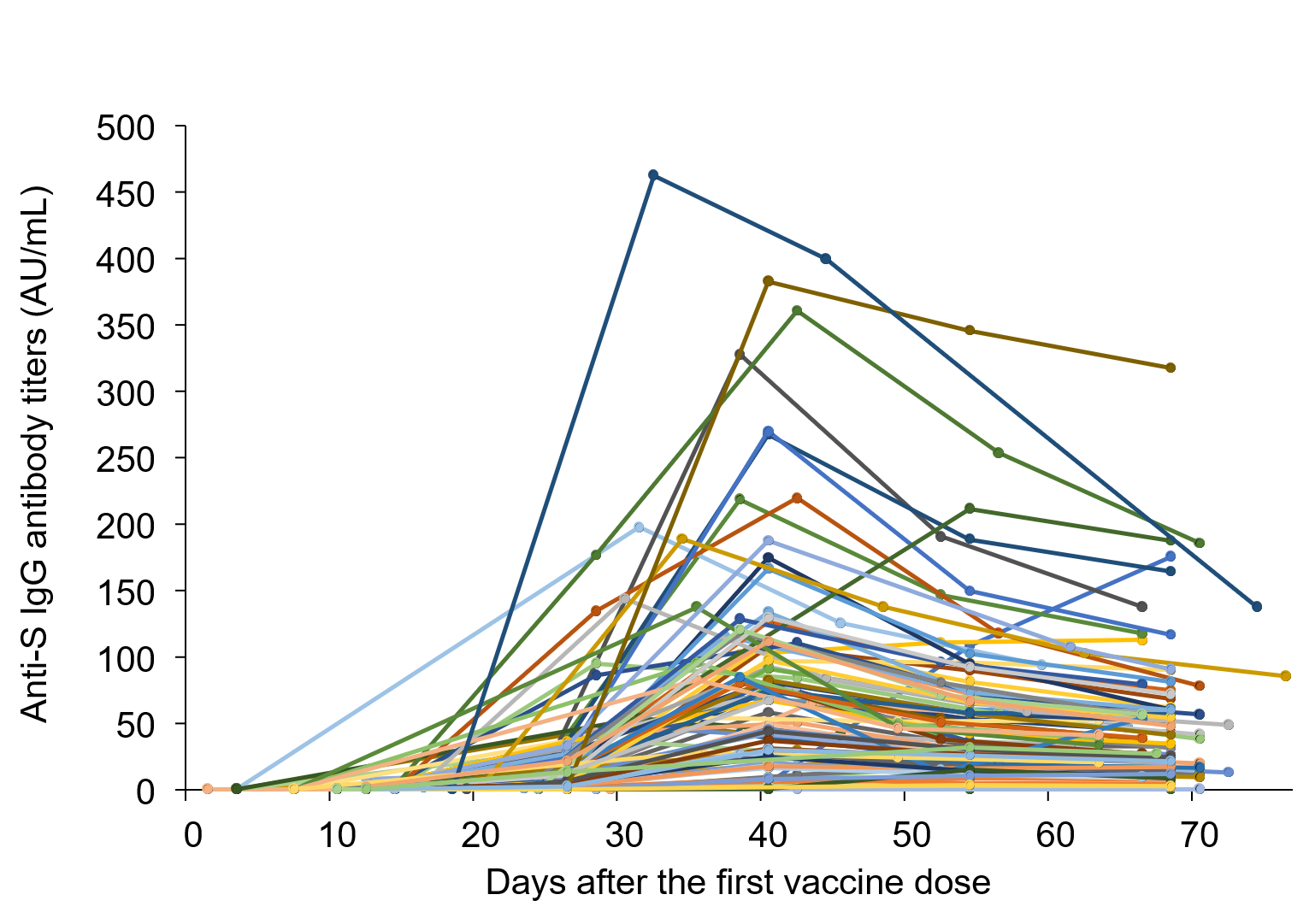


Additional file 3-b. Changes in anti-S IgG antibody titers from the day of the second dose


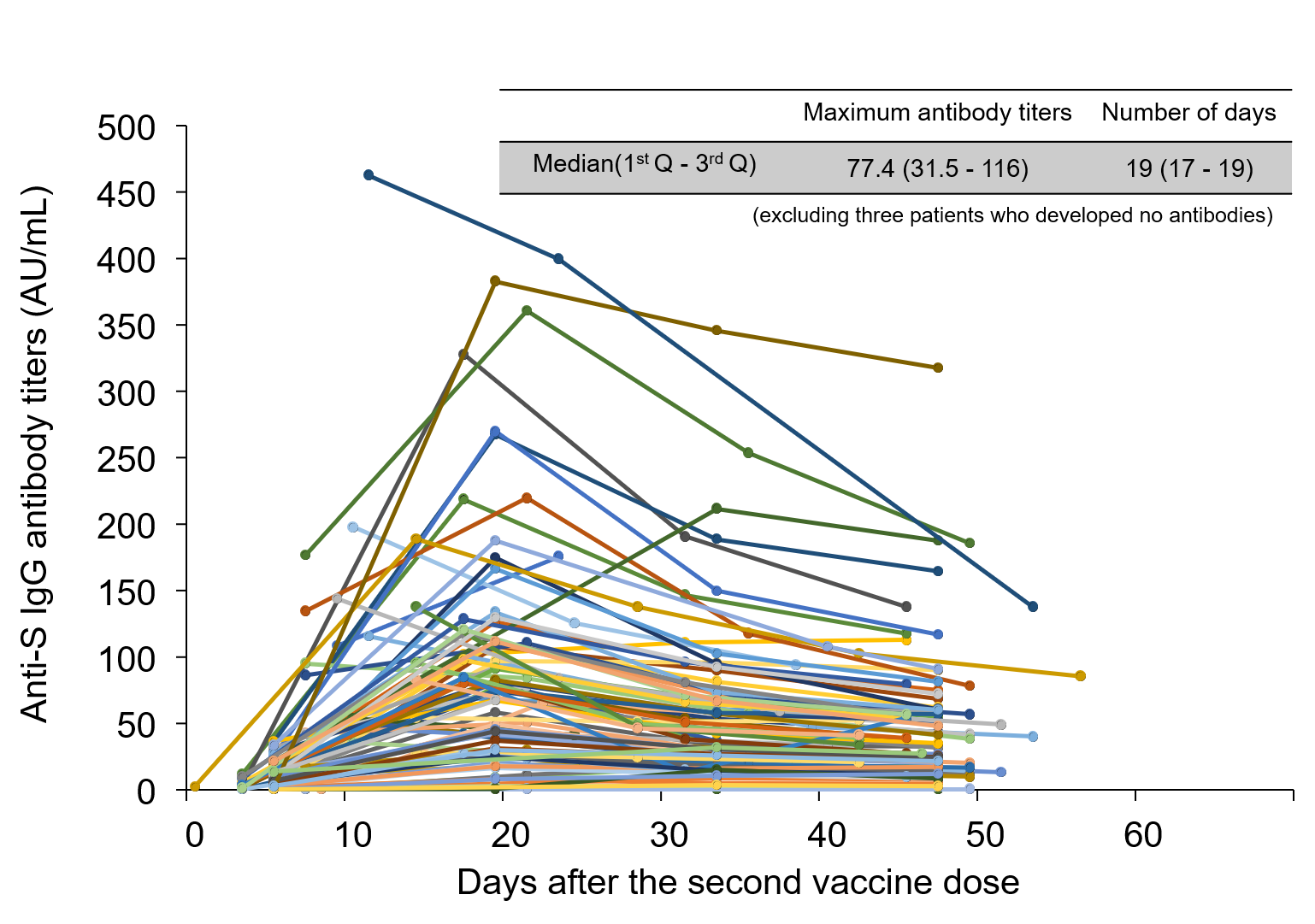

Supplement: Supplementary file 3 — Additional file 3. a. Changes in anti-S IgG antibody titers from the day of the first dose. b. Changes in anti-S IgG antibody titers from the day of the second dose. [file 12879_2022_7809_MOESM3_ESM.docx]
