# Supplementary material for: Changes in spike protein antibody titer over 90 days after the second dose of SARS-CoV-2 vaccine in Japanese dialysis patients
Source: BMC Infect Dis. 2022 Nov 14;22:852. doi: 10.1186/s12879-022-07809-1 (PMC9661455; doi:10.1186/s12879-022-07809-1)
Supplement: Supplementary file 4 — Additional file 4. a Correlation with maximum spike protein antibody titers. b. Maximum spike protein antibody titers by baseline patient characteristics. b. Maximum spike protein antibody titers by baseline patient characteristics. c. Factorial analysis of maximum spike protein antibody titers. d. Factorial analysis of log-maximum spike protein antibody titers. [file 12879_2022_7809_MOESM4_ESM.docx]

Additional file 4-a. Correlation with maximum spike protein antibody titers

| Item | n | ρ | p |
| --- | --- | --- | --- |
| Height (cm) | 96 | 0.189 | 0.065 |
| Body weight (kg) | 96 | 0.059 | 0.569 |
| Body Mass Index (kg/m^2^) | 96 | -0.061 | 0.553 |
| History of dialysis (years) | 96 | 0.159 | 0.121 |
| Creatinine Index | 96 | 0.280 | 0.006 * |
| C-reactive protein (mg/dL) | 96 | 0.023 | 0.823 |
| HbA1c (NGSP) (%) | 44 | 0.060 | 0.697 |
| PTH-intact (pg/mL) | 96 | 0.104 | 0.313 |
| Glycated albumin (%) | 44 | -0.040 | 0.794 |
| Ferritin (ng/mL) | 96 | -0.086 | 0.406 |
| Geriatric Nutritional Risk Index | 96 | 0.144 | 0.160 |
| Kt/V (shinzato) | 96 | 0.071 | 0.489 |
| Protein catabolism rate (g/kg/day) | 96 | 0.109 | 0.291 |
| Transferrin saturation (%) | 96 | -0.017 | 0.868 |
| Pre-dialysis albumin (g/dL) | 96 | 0.226 | 0.027 * |
| Clear space ratio (%) | 96 | 0.070 | 0.495 |
| Post-dialysis albumin (g/dL) | 96 | 0.142 | 0.166 |
| Age (years) | 96 | -0.168 | 0.101 |

*p<0.05 Spearman’s test

(Unless otherwise specified, the last pre-dialysis values before the first vaccine dose are shown.)

Additional file 4-b. Maximum spike protein antibody titers by baseline patient characteristics

| Item | | n | Median(1stQ,3rdQ) | p |
| --- | --- | --- | --- | --- |
| Sex | Male | 72 | 767.6 (407.9, 1196.4) | 0.165 |
|  | Female | 24 | 468.6 (222.9, 1059.8) |  |
| Diabetes | Applicable | 40 | 752 (248.9, 1160.9) | 0.908 |
|  | Not applicable | 56 | 728.7 (342.4, 1143.4) |  |
| Underlying disease: Nephrosclerosis | Applicable | 25 | 733.1 (313, 1059.8) | 0.576 |
|  | Not applicable | 71 | 742.8 (360.1, 1239.7) |  |
| Underlying disease: Chronic glomerulonephritis | Applicable | 16 | 647 (447.9, 1081.7) | 0.949 |
|  | Not applicable | 80 | 767.1 (277.7, 1143.4) |  |
| ≥60 years | Applicable | 65 | 639.7 (278, 1108.4) | 0.262 |
|  | Not applicable | 31 | 788.5 (360.1, 1225.1) |  |
| ≥65 years | Applicable | 56 | 596 (277.7, 1062.2) | 0.190 |
|  | Not applicable | 40 | 797.3 (374.5, 1247) |  |
| ≥70 years | Applicable | 49 | 493 (278, 934.4) | 0.042 † |
|  | Not applicable | 47 | 806 (360.1, 1302.9) |  |
| ≥75 years | Applicable | 39 | 639.7 (312, 997.1) | 0.260 |
|  | Not applicable | 57 | 788.5 (331.4, 1234.9) |  |

†p<0.05 Wilcoxon rank sum test

Supplementary material 4-c. Factorial analysis of maximum spike protein antibody titers

(n=96)

| Variables | Partial regression coefficient | Standard error | Standard partial regression coefficient | P-value |
| --- | --- | --- | --- | --- |
| Sex (1: male, 0: female) | 21.231 | 205.841 | 0.015 | 0.918 |
| ≥70 years | -283.393 | 170.939 | -0.162 | 0.101 |
| Body Mass Index (kg/m^2^) | -18.732 | 14.343 | -0.360 | 0.195 |
| Duration of dialysis (years) | -6.527 | 18.241 | -0.039 | 0.721 |
| Pre-dialysis Creatinine Index | 6.219 | 3.861 | 0.467 | 0.111 |
| Pre-dialysis albumin (g/dL) | 259.263 | 131.391 | 0.764 | 0.052 |

N.S. Multiple regression analysis.

Conditions predisposing to increased antibody titers: None

Accuracy of the regression formula: Adjusted R^2^=0.550

Significance of the regression formula: p<0.001

Shapiro-Wilk test of normality of residual error of the multiple regression analysis: p<0.001

The normality of residual error was rejected in the Shapiro-Wilk test.

Supplementary material 4-d. Factorial analysis of log-maximum spike protein antibody titers

(n=96)

| Variables | Partial regression coefficient | Standard error | Standard partial regression coefficient | P-value |
| --- | --- | --- | --- | --- |
| Sex (1: male, 0: female) | 0.215 | 0.128 | 0.067 | 0.095 |
| ≥70 years | -0.045 | 0.106 | -0.012 | 0.671 |
| Body Mass Index (kg/m^2^) | -0.001 | 0.009 | -0.008 | 0.914 |
| Duration of dialysis (years) | 0.002 | 0.011 | 0.006 | 0.851 |
| Pre-dialysis Creatinine Index | 0.005 | 0.002 | 0.170 | 0.037 * |
| Pre-dialysis albumin (g/dL) | 0.587 | 0.082 | 0.772 | <0.001 * |

Accuracy of the regression formula: Adjusted R^2^=0.968

Significance of the regression formula: p<0.001

Shapiro-Wilk test of normality of residual error of the multiple regression analysis: p=0.008

The normality of residual error was rejected in the Shapiro-Wilk test.
