# Supplementary material for: Changes in spike protein antibody titer over 90 days after the second dose of SARS-CoV-2 vaccine in Japanese dialysis patients
Source: BMC Infect Dis. 2022 Nov 14;22:852. doi: 10.1186/s12879-022-07809-1 (PMC9661455; doi:10.1186/s12879-022-07809-1)
Supplement: Supplementary file 5 — Additional file 5. a. Correlation with spike protein antibody titers at 90 days. b. Spike protein antibody titers at 90 days by baseline patient characteristics. c. Factorial analysis of spike protein antibody titers at 90 days. [file 12879_2022_7809_MOESM5_ESM.docx]

Additional file 5-a. Correlation with spike protein antibody titers at 90 days

| Item | n | ρ | p |
| --- | --- | --- | --- |
| Height (cm) | 81 | -0.024 | 0.830 |
| Body weight (kg) | 81 | -0.145 | 0.197 |
| Body Mass Index (kg/m^2^) | 81 | -0.194 | 0.082 |
| History of dialysis (years) | 81 | 0.135 | 0.230 |
| Creatinine Index | 81 | 0.148 | 0.186 |
| C-reactive protein (mg/dL) | 81 | -0.029 | 0.794 |
| HbA1c (NGSP) (%) | 36 | 0.215 | 0.208 |
| PTH-intact (pg/mL) | 81 | 0.078 | 0.488 |
| Glycated albumin (%) | 36 | 0.153 | 0.372 |
| Ferritin (ng/mL) | 81 | 0.061 | 0.589 |
| Geriatric Nutritional Risk Index | 81 | 0.048 | 0.674 |
| Kt/V (shinzato) | 81 | 0.213 | 0.056 |
| Protein catabolism rate (g/kg/day) | 81 | 0.092 | 0.413 |
| Transferrin saturation (%) | 81 | 0.106 | 0.345 |
| Pre-dialysis albumin (g/dL) | 81 | 0.178 | 0.113 |
| Clear space ratio (%) | 81 | 0.211 | 0.059 |
| Post-dialysis albumin (g/dL) | 81 | 0.169 | 0.131 |
| Age (years) | 81 | -0.145 | 0.196 |

N.S. Spearman’s test

(Unless otherwise specified, the last pre-dialysis values before the first vaccine dose are shown.)

Additional file 5-b. Spike protein antibody titers at 90 days by baseline patient characteristics

| Item | | n | Median(1stQ,3rdQ) | p |
| --- | --- | --- | --- | --- |
| Sex | Male | 60 | 201.5 (106.8, 337) | 0.467 |
|  | Female | 21 | 322 (115, 432) |  |
| Diabetes | Applicable | 32 | 181 (85.2, 352) | 0.385 |
|  | Not applicable | 49 | 229 (130, 375) |  |
| Underlying disease: Nephrosclerosis | Applicable | 24 | 193 (127.5, 321.3) | 0.587 |
|  | Not applicable | 57 | 242 (106, 384) |  |
| Underlying disease: Chronic glomerulonephritis | Applicable | 12 | 331 (234.3, 362.3) | 0.245 |
|  | Not applicable | 69 | 195 (106, 388) |  |
| ≥60 years | Applicable | 54 | 201.5 (98.3, 387) | 0.417 |
|  | Not applicable | 27 | 242 (164.5, 342.5) |  |
| ≥65 years | Applicable | 47 | 200 (101.2, 386) | 0.424 |
|  | Not applicable | 34 | 242 (162.3, 350.3) |  |
| ≥70 years | Applicable | 41 | 183 (95.4, 341) | 0.129 |
|  | Not applicable | 40 | 242.5 (148.8, 402) |  |
| ≥75 years | Applicable | 34 | 155 (98.3, 339.5) | 0.122 |
|  | Not applicable | 47 | 243 (137.5, 412.5) |  |

N.S. Wilcoxon rank sum test

Additional file 5-c. Factorial analysis of spike protein antibody titers at 90 days

(n=81)

| Variables | Partial regression coefficient | Standard error | Standard partial regression coefficient | P-value |
| --- | --- | --- | --- | --- |
| Age (years) | -1.573 | 2.374 | -0.264 | 0.510 |
| Sex (1: male, 0: female) | -72.547 | 82.751 | -0.150 | 0.383 |
| Body Mass Index (kg/m^2^) | -10.171 | 6.651 | -0.583 | 0.130 |
| Duration of dialysis | 5.161 | 8.484 | 0.079 | 0.545 |
| Pre-dialysis Kt/V (shinzato) | -558.167 | 468.708 | -1.931 | 0.237 |
| Pre-dialysis clear space ratio | 22.198 | 13.693 | 3.519 | 0.109 |

N.S. Multiple regression analysis.

Conditions predisposing to increased antibody titers: None

Accuracy of the regression formula: Adjusted R^2^=0.496

Significance of the regression formula: p<0.001

Shapiro-Wilk test of normality of residual error of the multiple regression analysis: p<0.001

The normality of residual error was rejected in the Shapiro-Wilk test.
