# Supplementary material for: Changes in spike protein antibody titer over 90 days after the second dose of SARS-CoV-2 vaccine in Japanese dialysis patients
Source: BMC Infect Dis. 2022 Nov 14;22:852. doi: 10.1186/s12879-022-07809-1 (PMC9661455; doi:10.1186/s12879-022-07809-1)

Additional file 6. Predictive performance of spike protein antibody titers at 30 days for spike protein antibody titers >137 at 90 days

AUC:0.925, Cut-off value: 313 (excluding values converted from anti-S IgG antibody titers)


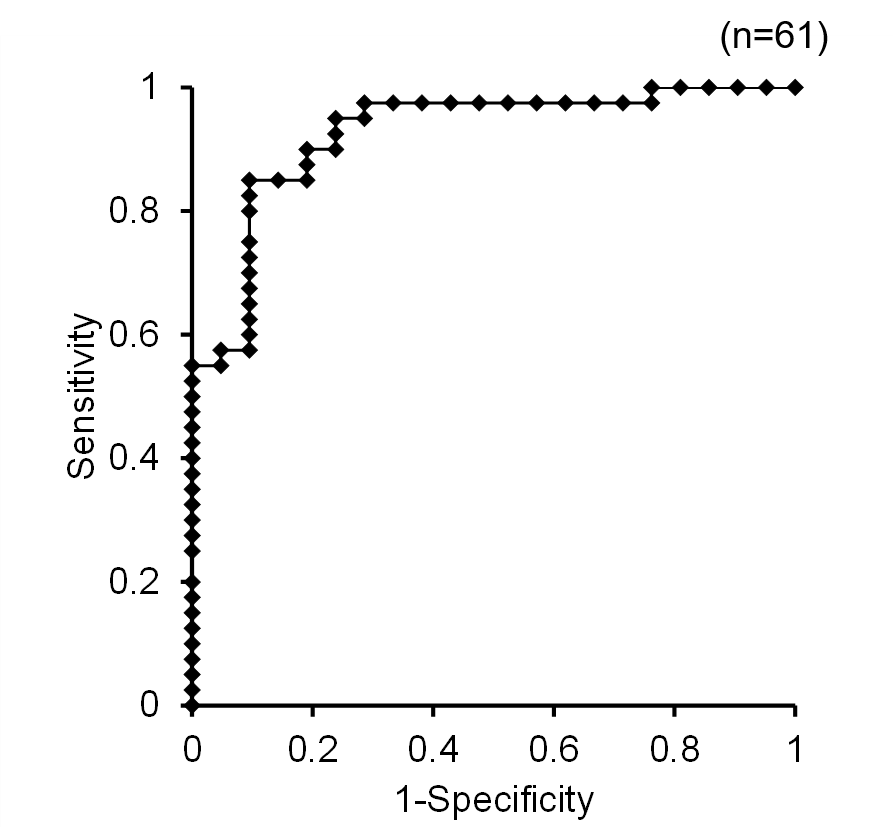

Supplement: Supplementary file 6 — Additional file 6. Predictive performance of spike protein antibody titers at 30 days for spike protein antibody titers >137 at 90 days. [file 12879_2022_7809_MOESM6_ESM.docx]
