# Supplementary material for: Changes in spike protein antibody titer over 90 days after the second dose of SARS-CoV-2 vaccine in Japanese dialysis patients
Source: BMC Infect Dis. 2022 Nov 14;22:852. doi: 10.1186/s12879-022-07809-1 (PMC9661455; doi:10.1186/s12879-022-07809-1)
Supplement: Supplementary file 7 — Additional file 7. a. Correlation between anti-S IgM antibody titers and anti-S IgG antibody titers. b. Correlation between anti-S IgM antibody titers and anti-S IgG antibody titers (correlation between the maximums of both titers). [file 12879_2022_7809_MOESM7_ESM.docx]

Additional file 7-a. Correlation between anti-S IgM antibody titers and anti-S IgG antibody titers


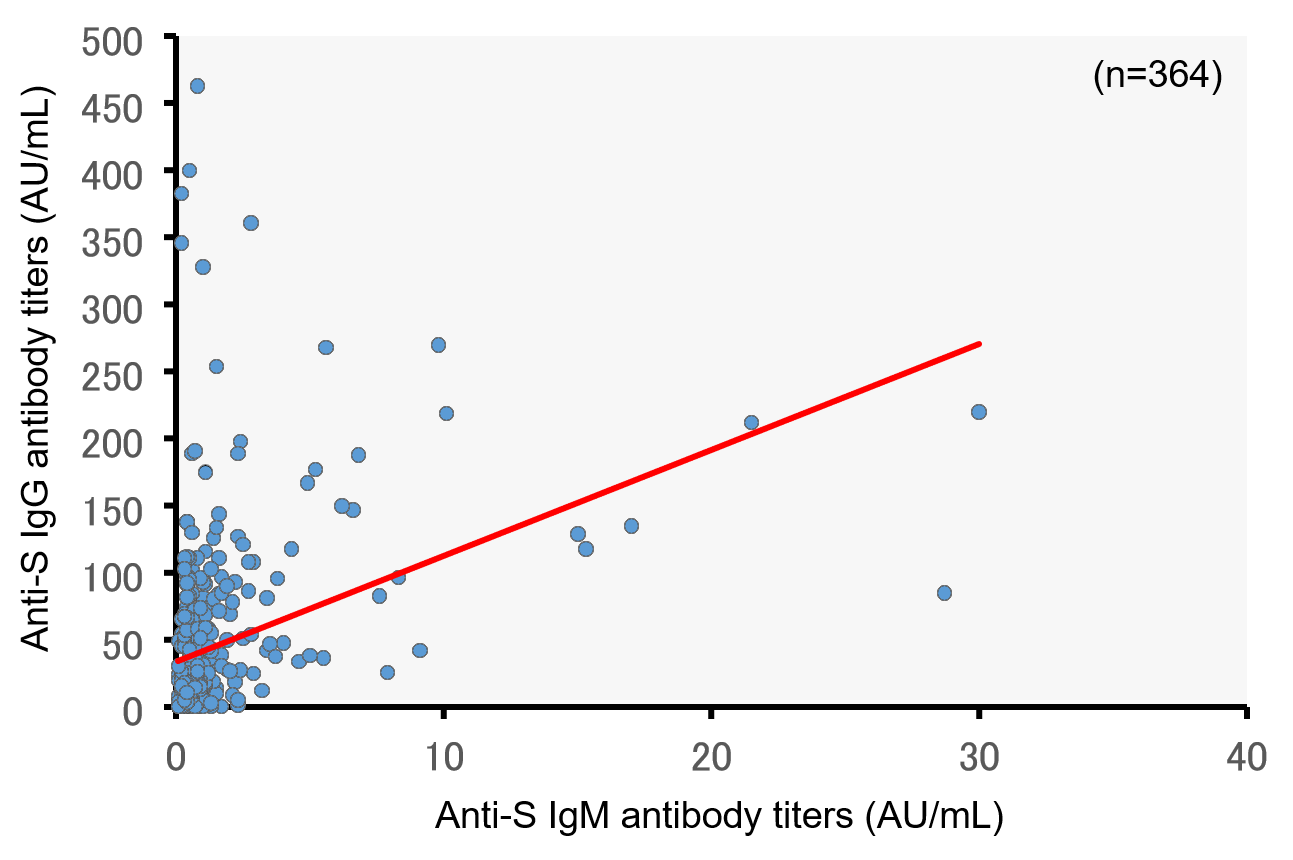


ρ=0.674, p<0.001 Spearman’s test.

Additional file 7-b. Correlation between anti-S IgM antibody titers and anti-S IgG antibody titers (correlation between the maximums of both titers)


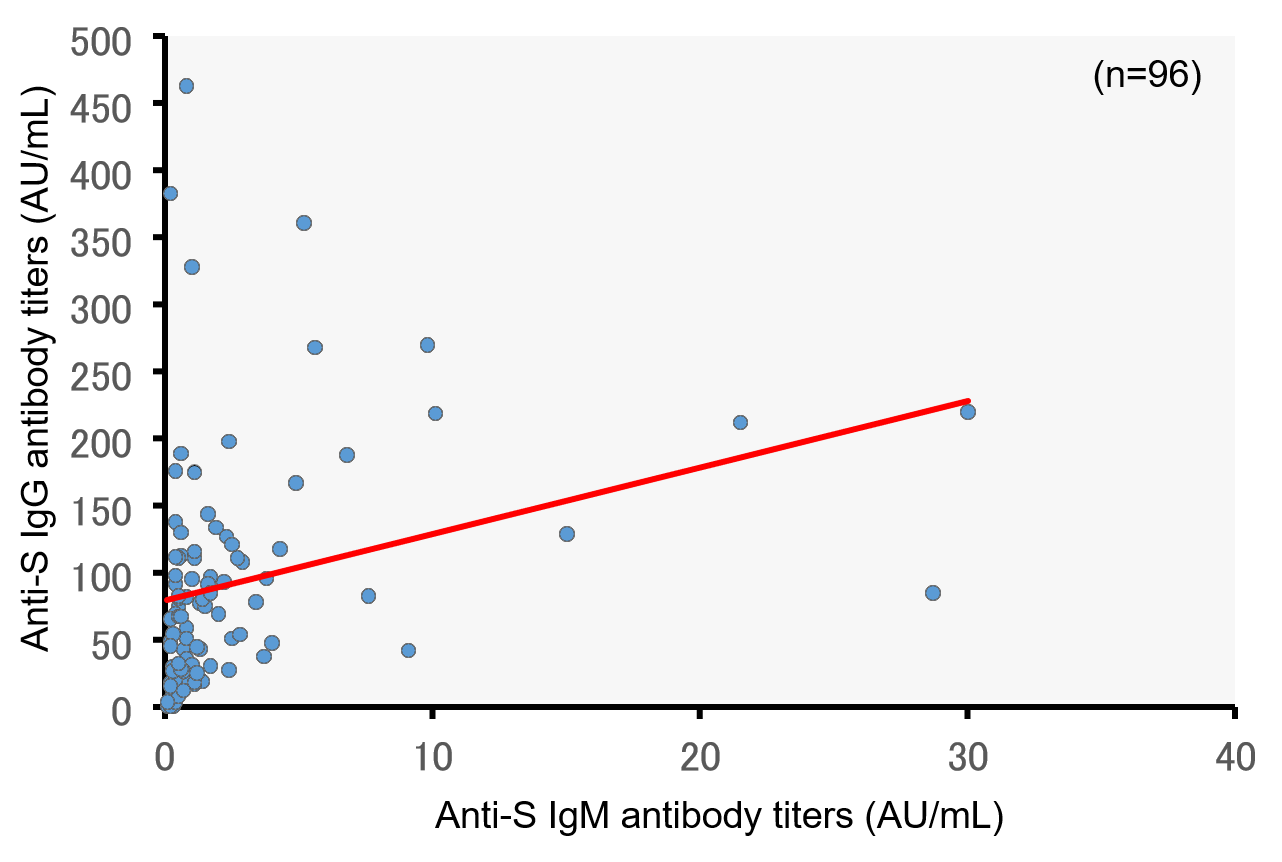


ρ=0.458, p<0.001 Spearman’s test
